# Brain-Derived Neurotrophic Factor and Immune Cells in Osteoarthritis, Chronic Low Back Pain, and Chronic Widespread Pain Patients: Association with Anxiety and Depression

**DOI:** 10.3390/medicina57040327

**Published:** 2021-04-01

**Authors:** Dominique Josephine Dimmek, Christoph Korallus, Sabine Buyny, Gutenbrunner Christoph, Ralf Lichtinghagen, Roland Jacobs, Boya Nugraha

**Affiliations:** 1 Department of Rehabilitation Medicine, Hannover Medical School, Carl-Neuberg-Str.1, 30625 Hannover, Germany; Dimmek.Dominique@mh-hannover.de (D.J.D.); Korallus.Christoph@mh-hannover.de (C.K.); Gutenbrunner.Christoph@mh-hannover.de (G.C.); 2 Department of Rheumatology and Clinical Immunology, Hannover Medical School, Carl-Neuberg-Str.1, 30625 Hannover, Germany; Buyny.Sabine@mh-hannover.de (S.B.); Jacobs.Roland@mh-hannover.de (R.J.); 3 Institute of Clinical Chemistry, Hannover Medical School, Carl-Neuberg-Str.1, 30625 Hannover, Germany; Lichtinghagen.Ralf@mh-hannover.de

**Keywords:** osteoarthritis, chronic low back pain, chronic widespread pain, BDNF, anxiety, depression, immune cells

## Abstract

*Background and Objectives*: Musculoskeletal dysfunction can induce several types of chronic pain syndromes. It is of particular interest to elucidate the pathomechanism of different forms of chronic pain. It is possible that patients who have developed chronic widespread pain (CWP) may endure different pathomechanisms as compared to those who suffer from local pain (osteoarthritis, OA) and regional pain (chronic low back pain, cLBP), especially with regard to pain regulation and its related biomediators. The aim of this study was to determine the differences in pathomechanisms among these patients by measuring pain-related biomediators, particularly brain-derived neurotrophic factor (BDNF). Additionally, subpopulations of immune cells were determined in parallel. *Materials and Methods*: Patients and healthy subjects (HSs) were recruited (age and gender-matched). BDNF was measured from serum samples of patients and HSs and the data of body composition parameters were recorded. Additionally, both patients and HSs were asked to fill in questionnaires related to pain intensity, anxiety, and depression. *Results*: Our results highlight that the levels of both free and total BDNF are significantly lower in pain patients compared to HSs, with *p* values of 0.041 and 0.024, respectively. The number of CD3^−^ CD56_bright_ natural killer (NK) cells shows significant differences between the groups. Comparing all chronic pain patients with HSs reveals a significantly lower number of CD4^+^ CD8^+^ T cells (*p =* 0.031), CD3^−^ CD56_bright_ NK cells (*p =* 0.049) and CD20^+^ CD3^−^ cells (*p* = 0.007). *Conclusions*: To conclude, it seems that a general conformity between the pathomechanisms of different chronic pain diseases exists, although there are unique findings only in specific chronic pain patients.

## 1. Introduction

Pain is considered chronic when it lasts for more than three to six months after the healing of the original injury or recurs repeatedly [1]. Chronic pain may result from the persistence of pathological conditions that activate the nociceptive system [2]. Recently, the International Association of Pain proposed a systematic classification of chronic pain. It is divided into chronic primary pain and chronic secondary pain syndromes [3]. Chronic widespread pain and chronic primary musculoskeletal pain are examples of chronic primary pain. Chronic pain patients frequently show other symptoms, such as depression, anxiety, fatigue, and sleep disturbance, in addition to suffering from pain [4]. They also show reduced physical activity [4]. Taken together, these symptoms may lead to disability and decreased quality of life [5].

Osteoarthritis (OA) is one of the most prevalent chronic pain disorders [6]. The prevalence of OA is likely to increase because of aging of the population and obesity [6]. OA is a chronic progressive degenerative joint disease that involves not only articular cartilage but also synovial and subchondral bone as well as the surrounding muscles and ligaments. It is characterized by cartilage destruction, subchondral bone sclerosis, and osteophyte or cyst formation [7]. Knee osteoarthritis is a major cause of severe pain, joint stiffness, limited motion, and disability [8]. It is also hypothesized that it can be caused by abnormal excitability in peripheral and central pathways [9].

Low back pain is defined as “pain, muscle tension or stiffness localized below the costal margin and above the inferior gluteal folds, with or without leg pain (sciatica)” [10]. The likelihood of having any back pain in a year varies between 58% and 66%, which makes low back pain a highly prevalent burden [11,12]. There are many types of treatments for chronic low back pain (cLBP), including drugs, exercise [13], patient education [14], and yoga [15]. However, the results are still conflicting, which could be a result of a lack of information about the mechanism in cLBP [16]. Further elucidating the pathomechanism of cLBP might, therefore, mediate an effective treatment for cLBP patients.

Chronic widespread pain (CWP), such as fibromyalgia syndrome (FMS), is a disorder of pain regulation with central sensitization [17]. CWP is a common symptom in the community, with a prevalence of one in ten individuals globally [18]. In addition to pain, CWP patients reveal other symptoms, such as fatigue, psychological distress, and somatic symptoms [18]. Clinical symptoms of CWP conditions include pain, stiffness, subjective weakness, and muscle fatigue [18].

Central sensitization is a form of maladaptive neuroplasticity underlying many chronic pain disorders, including neuropathic pain, fibromyalgia [19], some forms of osteoarthritis [20], and low back pain [21]. Brain-derived neurotrophic factor (BDNF) is a member of neurotrophins (NTs), which are essential for the normal development of the vertebrate nervous system and play a role in neuroplasticity [22]. NTs are produced inter alia and released by astrocytes [23]; immune cells, including lymphocytes mast cells; activated monocytes; T and B cells [24]. Some NTs, especially BDNF and nerve growth factor (NGF), play a significant role in nociception in different chronic pain diseases [19,24,25]. Inflammation can be associated with chronic pain; however, the level of pro- and anti-inflammatory cytokines is still inconclusive [26]. Although there is a link between inflammation and BDNF [22], it has been reported that BDNF plays a role in sleep deprivation and depression [22]. These symptoms are commonly found in chronic pain patients [4].

As afore-mentioned, chronic musculoskeletal pain patients suffer from psychological symptoms, sleep deprivation, and other mental-related issues [17,27]. Some studies report BDNF in different chronic pain patients, including fibromyalgia [17,28,29,30] and osteoarthritis [31]. However, there is a lack of reports on BDNF in cLBP. We hypothesized that patients who have developed CWP may potentially endure different pathomechanisms compared to those who only suffer from local (OA) and regional pain (cLBP), especially with respect to pain regulation and its related biomediators [32,33]. Additionally, localized pain may be caused by peripheral mechanical strain and tissue irritation [32]. As prescribed in CWP, central sensitization might be involved, which leads to an overall lowering of the pain threshold [34]. Therefore, this study aimed at the determination of pain-related biomediators and linked them to mood-related symptoms, which may help us to differentiate these types of patients. BDNF is our molecule of interest, as it has been studied in different types of chronic pain patients. Additionally, subpopulations of immune cells were determined in parallel.

## 2. Materials and Methods

This study was a cross sectional study and performed according to the principles of Helsinki (1961). It was approved by the Ethics Committee of Hannover Medical School (No. 6554). Setting: University hospital.

### 2.1. Participants

#### 2.1.1. Patients

Inclusion. To be included, all patients (male and female) should have a history of pain for at least three months. This study focused on three different types of chronic pain patients, with a score of a minimum of ≥4 on the Visual Analogue Scale (VAS) during the last week before the day of recruitment. Patients were 18 to 70 years of age and understood the German language. The patients had to have met the criteria of the diagnosis of OA (knee(s) or hip(s)), cLBP, or CWP. The diagnosis of OA [35,36] and cLBP [37] were assessed according to patients’ history and medical records by experienced physicians at the Department of Rehabilitation Medicine, Hannover Medical School. The CWP had to match the definition of Manchester Widespread Pain [38].

Exclusion. Patients were excluded in cases of having a comorbidity, such as cancer, heart failure, major depressive disorder, acute and inflammatory diseases, a recent injury, symptomatic arteriosclerosis, or diabetes. Specific exclusion criteria were radiculopathy, sciatica, and any indication of immediate surgery, autoimmune and infection joint, and acute spine disease. Pregnant and breast-feeding female patients were excluded, as well.

Patients were not allowed to take any pain medication within two days before blood drawing. However, in emergency cases, they were allowed to take emergency medicine.

Patients were recruited by using advertisement in the local newspaper, following telephone screenings. Additionally, patients were also recruited from outpatients of the Department of Rehabilitation Medicine and other departments of Hannover Medical School, Hannover, Germany.

#### 2.1.2. Healthy Subjects (HSs)

Most HSs were staff of Hannover Medical School, Hannover, Germany. They were examined at the Department of Rehabilitation Medicine. We recruited HSs that matched the chronic pain patient group by gender and age (±2 years). All HSs should be particularly free from chronic pain and other exclusion criteria that were applied for patients.

### 2.2. Outcome Parameters

#### 2.2.1. Biological Mediators

BDNF. Fasting blood samples (30 mL each) from an arm vein were drawn in the morning between 7:00 and 10:00 a.m. Samples were allowed to clot before being centrifuged at 1500× *g* for 15 min. Serum samples were stored at −80 °C at the Biobank of Hannover Medical School until analyzed. Free and total BDNF were measured by using conventional sandwich ELISA in serum samples (Quantikine, R&D System). The quantification was performed against a standard curve as provided by the manufacturer in a plate reader.

Phenotyping of peripheral blood lymphocytes by flow cytometry. Phenotyping of lymphocytes was performed by incubating 200 µL of freshly drawn heparinized whole blood with antibodies according to the manufacturers’ recommendations in 5 mL tubes. The following antibodies were used for this study: CD3 PE-Cy7, CD4 FITC, CD8 PB, CD14 BV510, CD20 APC, CD45 APC-Cy, and CD56 PE. 7 AAD was used for live cell gating (Biolegend, London, UK). Corresponding isotype-matched antibodies were used as controls. All antibodies were purchased from Biolegend. After 20 min incubation, 2 mL of lysis solution (Biolegend) was added to each tube. Then, 10 min later, tubes were centrifuged (3 min at 400× *g*), and cells were washed three times with 2 mL of PBS/BSA (3 min at 400× *g*). Finally, cells were resuspended in 250 mL PBS/BSA and subjected to flow cytometry analysis on a FACSCanto II (Becton Dickinson, Heidelberg, Germany) by gating on live CD45+ singlet lymphocytes and acquiring 5 × 104 to 1 × 105 events per sample in the combined gate. Offline data analysis was performed by using FSC Express V6 software (Denovo, Pasadena, CA, USA). Absolute cell counts were calculated on the basis of WBC counts obtained from the Department of Hematology at Hannover Medical School.

#### 2.2.2. Questionnaires

Patients and HSs were asked to fill in several questionnaires, including pain intensity, which was measured by using VAS, and the depression score, which was measured by using the Hospital Anxiety and Depression Scale (HADS) [39]. Additionally, body composition parameters were measured by using the InBody machine (InBody 230; Model MW160, Korea). This machine provides information such as body weight, body mass index (BMI), skeletal muscle mass (SMM), body fat mass (BFM), body water mass (BWM), fat free mass (FFM), body fat percentage (BFP), and waist–hip ratio (WHR).

### 2.3. Statistical Analyses

The normality of the data was checked by using the Shapiro–Wilk test. An ANOVA or Kruskal–Wallis test (depending on the data distribution) was used to compare the significance of body composition parameters, BDNF, immune cell phenotype, and HADS (anxiety and depression) followed by a post hoc test with Bonferroni correction (set up in the SPSS 26 (IBM, New York, NY, USA)).

A Student’s t-test or Mann–Whitney U test was used to compare the free and/or total BDNF between HSs and patients depending on data distribution. The mean imputation method was used for handling missing values. Statistical analyses were performed by using SPSS version 26 (IBM, New York, NY, USA). Results were considered statistically significant at *p* < 0.05. Subgroup analysis was performed according to the anxiety and depression score. Explorative statistics analyses, such as the correlation of biological mediators and body composition parameters, were also performed.

### 2.4. Sample Size Calculation

The most comparable research was published by Laske et al. [30], who analyzed the BDNF levels in FMS patients (supposed as CWP patients) and HSs. Thus, that result was used for sample size calculation in our research. This study was planned for a continuous response variable from independent control and experimental subjects with one HS per patient, matched by gender and age (±2 years). In a previous report [30], the response within each subject group was normally distributed with a standard deviation of 3.1. In this study, we had four groups, using a sample size calculation for the ANOVA test, with the lowest mean of 16.8 in HSs and 19.6 in CWP patients. We assumed that the means of the other two groups would be equal to the grand mean (18.2). Therefore, we would need a total sample size of 144 (36 subjects/group) to be able to reject the null hypothesis that the population means of all groups were equal with a probability (power) of 0.9. The type I error probability associated with this test of the null hypothesis was 0.05.

## 3. Results

### 3.1. Patients Recruitment

**Figure 1:**
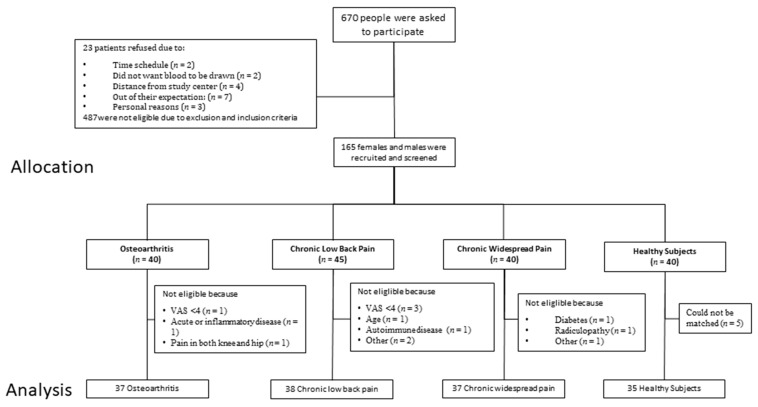
Flow chart recruitment.

In total, 670 participants were asked to participate in this study. Due to different reasons, such as exclusion criteria, time schedule, and the distance from participants’ addresses, final data from 37 patients with OA, 38 patients with cLBP, 37 patients with CWP, and 35 HSs were analyzed (Figure 1). None of the participants needed to resort to taking emergency medication.

### 3.2. Age, Sex, and Body-Related Composition Parameters

Table 1 demonstrates the mean values of age, sex distribution, and body-related parameters. There were no significant differences regarding age. Sex distributions were significantly different between groups. It is important to note that all body-related parameters were significantly different between groups.

Significant differences concerning the height of all our patients were found between CWP patients and OA patients (*p =* 0.001) as well as between CWP patients and cLBP patients (*p =* 0.043). Patients with OA showed significant differences when comparing their BW with HSs (*p =* 0.001), with cLBP patients (*p =* 0.025) and CWP patients (*p =* 0.021). The BMI revealed significant differences between HSs and OA patients (*p =* 0.001) and HSs and patients with CWP (0.025).

As expected, HSs had significantly less pain on the VAS compared to all chronic pain patients. Furthermore, OA patients showed significantly less pain compared to CWP patients (*p =* 0.001). OA patients had significantly higher SMM compared to all other groups (OA vs. HSs: *p =* 0.025; OA vs. cLBP: *p =* 0.014; OA vs. CWP: *p =* 0.01). BFM was significantly different between HSs and OA patients (*p =* 0.004) as well as HSs and CWP patients (*p =* 0.017). Regarding BWM, significant differences between OA patients and other groups were observed (OA vs. HSs: *p =* 0.018; OA vs. cLBP: *p =* 0.014; OA vs. CWP: *p =* 0.001). OA patients indicated significant differences from all groups concerning their FFM (OA vs. HSs: *p =* 0.018; cLBP vs. OA: *p =* 0.013; OA vs. CWP: *p* < 0.001). It was testified that our HSs had significantly less BFP than all our chronic pain patients (OA vs. HSs: *p =* 0.003; cLBP vs. HSs: *p =* 0.011; CWP vs. HSs: *p* < 0.001). In addition to this, the WHR of our HSs was significantly lower than in all of our other groups as well (OA vs. HSs: *p* < 0.001; cLBP vs. HSs: *p =* 0.009; CWP vs. HSs: *p =* 0.002).

### 3.3. Free and Total BDNF

**Figure 2:**
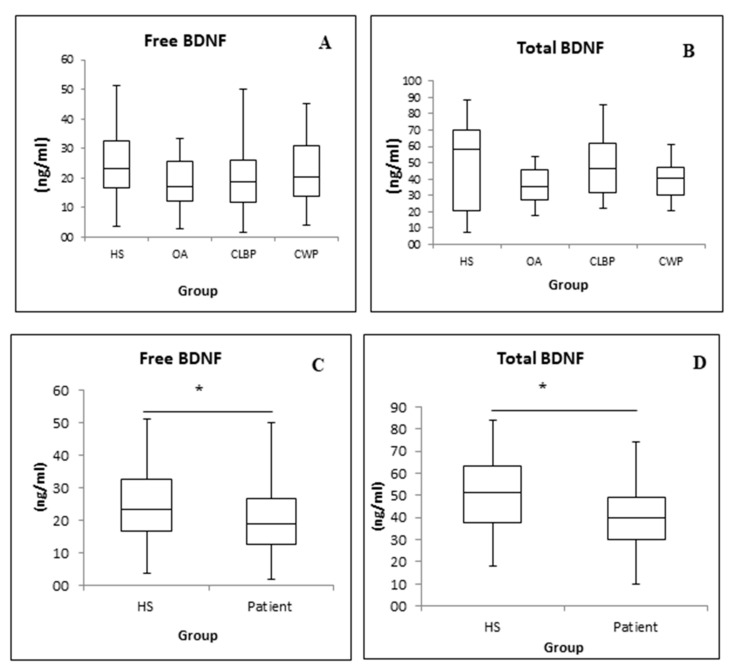
Free brain-derived neurotrophic factor (BDNF) (**A**) and total BDNF (**B**) in healthy subjects, osteoarthritis, chronic low back pain, and chronic widespread pain patients. Free BDNF (**C**) and total BDNF (**D**) in healthy subjects vs. cumulative patients * *p* < 0.05.

Figure 2 shows BDNF levels (free (2A) and total (2B)) in HSs and different patient groups. No significant differences were observed among groups. Significant differences were found when comparing the HSs and total pain patient group (comprising all patients) both in free BDNF (2C) and total BDNF (2D). The levels of both free and total BDNF were significantly lower in pain patients, with *p* values of 0.041 and 0.024, respectively.

### 3.4. Phenotypic Profiles of Immune Cells in Chronic Pain Patients

Several immune cells were also compared and analyzed (Table 2). Only CD3^−^ CD56_bright_ showed significant differences between the groups. A significant difference concerning CD3^−^ Cd56_bright_ immune cells was found when comparing HSs with OA (*p =* 0.009), cLBP (*p =* 0.023), and CWP (*p =* 0.002) patients.

**Figure 3:**
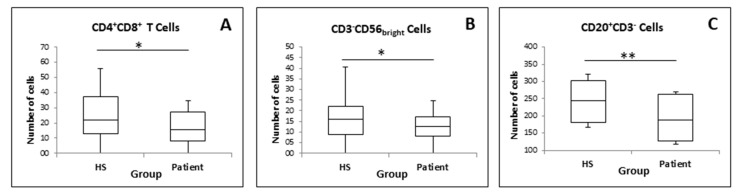
Immune cells between HSs and chronic pain patients: (**A**) CD4^+^ CD8^+^ T cells; (**B**) CD3^−^ CD56_bright_; (**C**) CD20^+^ CD3^−^. * *p* < 0.05; ** *p* < 0.01.

Comparing total pain patients (all patients) with HSs (Figure 3) revealed significant differences in CD4^+^ CD8^+^ T cells, double positive T (DPT) cells (*p =* 0.031), CD3^−^ CD56_bright_ natural killer cells (NK) (*p =* 0.049), and CD20^+^ CD3^−^ B cells (*p =* 0.007).

### 3.5. Anxiety and Depression

**Figure 4:**
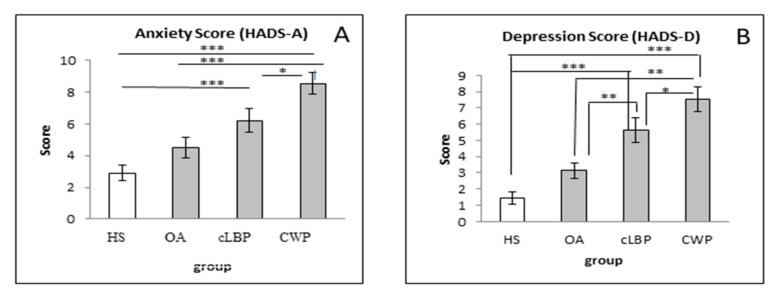
Level of anxiety and depression in HSs and OA, cLBP, and CWP patients; * *p* < 0.05; ** *p* < 0.01; *** *p* < 0.001. Levels of anxiety and depression were determined by using HADS-A (**A**) and HADS-D (**B**), respectively.

The results (Figure 4) showed significant differences between groups of patients with regard to the levels of both anxiety and depression. Interestingly, the levels of anxiety and depression in cLBP and CWP, but not OA patients, were significantly different from those in HSs.

Subgrouping the numbers of patients according to HADS-A and HADS-D scores revealed unexpected results (Table 3). More than 80% and 70% of OA and cLBP patients neither had anxiety nor depression. The prevalence of anxiety (HADS-A Score ≥ 11) in OA, cLBP, and CWP patients was 10.8%, 18.4%, and 35.10%, respectively. Meanwhile, the prevalence of depression (HADS-D Score ≥ 11) in OA, cLBP, and CWP patients was 5.0%, 18.4%, and 27.0%, respectively.

### 3.6. BDNF and Immune Cells Subgroup Analysis Based on Anxiety and Depression Score

Subgroup analysis based on the anxiety score is demonstrated in Table S1 (Supplementary). A significant difference was exclusively found in the level of free BDNF in OA patients. Post hoc analysis showed a significant difference between an anxiety score ≤7 and ≥11 (*p =* 0.023). There was no other statistical difference between BDNF and immune cells.

Supplementary Table S2 shows the levels of BDNF (free and total) and immune cell numbers according to the subgroups of depression score. A significant difference was exclusively found in the CD20^+^ CD3^−^ of cLBP patients. Furthermore, post hoc analysis showed a significant difference in the immune cell numbers of cLBP patients between a depression score ≤7 and ≥11 (*p =* 0.017), although after Bonferroni corrections, the level of significance was not reached (*p =* 0.052). There was no significant difference with regard to BDNF and immune cells in the cumulative group of patients (Supplementary Table S3).

### 3.7. Clinical Data and Biological Mediators

Table 4 shows the correlations of clinical data with biological mediators and/or other clinical parameters in all chronic pain patients. In OA patients, free and total BDNF revealed a significant negative correlation with BW. Interestingly, the depression score and CD3^−^ CD56_bright_ natural killer cell (NK) numbers yielded a significant negative correlation. In cLBP patients, anxiety and depression scores revealed positive correlations with pain intensity. In CWP patients, anxiety and depression scores revealed a positive correlation with pain intensity. Biological mediators did not show any significant correlations with clinical parameters in CWP patients.

## 4. Discussion

The aim of this study was to determine the pathomechanisms of different chronic pain patients, particularly by investigating BDNF.

Comparing OA, cLBP, and CWP patients and HSs, there were no significant differences concerning BDNF. Interestingly, comparing the collected data of all patients, the levels of free and total BDNF were significantly lower than in HSs (*p* < 0.05).

It is well established that the BDNF level is different in chronic pain patients and in people who suffer from major depression as compared to HSs [19,40]. For instance, in patients with OA [41], the BDNF level is lower compared to that in HSs, whereas it is higher in patients with fibromyalgia [29]. The latter report contradicts the findings that show no significant difference between fibromyalgia syndrome patients and healthy controls [42]. The differences among patient groups and the characteristics of patients could be the reasons [42]. However, our study supports the latter report. Concerning CWP, there are no specific studies focusing on CWP in general, but some studies in fibromyalgia have been recorded. The studies that showed an agreement with an increase in the BDNF level in fibromyalgia [17,29,30] only recruited female patients. Another study that reported no differences in BDNF levels recruited both female and male patients [42]. Although there are conflicting results concerning BDNF and gender, some studies support different levels of BDNF in different genders [43,44]. Therefore, further studies need to be performed in this group of patients with regard to gender difference. To the best of our knowledge, this is the first study that investigates BDNF in cLBP.

Seifert et al. [40] concluded that the body composition plays a role regarding the amount of BDNF. Body parameters such as BW, BMI, and BFP among all our chronic pain patients showed comparably higher data than our HSs, who showed a higher amount of BDNF. Further studies need to be performed, as Seifert et al. determined BDNF in only healthy people [40]. Among specific musculoskeletal diseases, such as OA, many studies have shown a correlation with obesity [45]. It seems that the higher these specific body composition parameters are, the lower the BDNF level is. Our results highlight that, since our patients are overweight and suffer from chronic musculoskeletal pain, their biological parameters may be affected.

Studies have shown that the production of BDNF can be stimulated by acute exercise [46,47]. Hence, there should be a correlation between body composition parameters and the level of BDNF. In this study, free and total BDNF showed a negative correlation with BW in patients with OA. BDNF is known to play a role in the control of energy balance and satiety, and further mutation in its gene can contribute to severe obesity [48].

BDNF can be divided into free and bounded with tyrosine kinase receptors, the total BDNF representing the sum of free BDNF and BDNF that binds to tropomyosin kinase receptors (Trk) [49]. In this study, BDNFwas measured as free and total. This study showed a similar pattern of both free and total BDNF in association with the characteristics of all groups of patients, particularly pain, depression, and anxiety. However, both free and total BDNF correlated significantly with the body weight of OA patients only. To the best of our knowledge, this is the first study that reports both free and total BDNF in chronic pain patients.

Our patients showed not only remarkably higher body composition parameters but also psychological problems compared to HSs. Mood disorders are more likely to develop in patients with chronic pain [50]. The study of Mundal et al. [51] illustrates that not only psychosocial factors but also lifestyle factors influence the chance of getting CWP, including anxiety and depression as well as a BMI < 18.5 kg/m^2^ or BMI > 25 kg/m^2^. We found that our patients had an average BMI of 27.68 ± 0.92 and that 35.1% of our CWP patients showed high scores of anxiety and 10% a high score of depression.

It is known that central sensitization can lead to intensified pain [52], and an increase in pain correlates with a reduction in quality of life [53]. Our patients with cLBP showed lower levels of both anxiety and depression compared to CWP patients. However, mood disorders correlate with pain, and given that CWP patients possess more regions affected by pain, it is possible that many CWP patients, therefore, suffer from higher pain intensity than those who only suffer from regional pain and local pain.

In addition to BDNF, we explored different types of immune cells in these groups of patients. Previous studies have shown significant differences in the CD3^+^ CD56^+^ NK cells between FMS patients and HSs [54]. In this study, only CD3^−^ CD56_bright_ NK cells showed a significant difference between groups. To our knowledge, there are no studies reporting CD3^−^ CD56_bright_ NK cells and free BDNF in patients with chronic low back pain. Additionally, we found that OA patients with depression showed a significant negative correlation with CD3^−^ CD56_bright_ NK cells. Therefore, the higher the level of depression, the lower the amount of these specific immune cells. To date, CD3^−^ CD56_bright_ NK cells have not been investigated in patients with osteoarthritis (in knees or hips) in combination with depression. However, there are studies researching the connection of depression with natural killer cells and other studies focusing on the amount of natural killer cells in osteoarthritis [55]. Majidi-Zolbanin et al. [56] found that patients suffering from major depressions show a reduced number of circulating NK cells and concluded that depression seems to be associated with a suppression of the immune system. Although not concerning OA and not specifically investigating CD3^−^ CD56_bright_ NK cells, but rather NK cells in general, there seems to be a connection between depression and NK cells. In a study of Wang et al. [57], natural killer cells were examined in patients with OA and HSs. They found that the CD56_bright_ CD16^−^ NK subset in OA patients was augmented and that CD56_bright_ CD16^−^ NK cells from OA patients had reduced regulatory functions.

Comparing all chronic pain patients to HSs, CD3^−^ CD56_bright_ NK cells revealed significant differences. It is known that patients suffering from cLBP or patients with musculoskeletal problems show a lower percentage of NK cells than asymptomatic subjects [58]. Additionally, in patients with FMS, the number of natural killer cells is lower compared to those without FMS [59].

Another significant difference was found in CD4^+^ CD8^+^cells and CD20^+^ CD3^−^ (B) cells while comparing all chronic pain patients with HSs. It is known that T lymphocytes not only have a promoting effect on chronic pain, but they also play a role in the transition from acute to chronic pain [60]. Interestingly, the research by Ziv et al. [61] has shown that T cells are required for BDNF expression. Since B and T cells were decreased in our pain patients compared to HSs, this may be why the BDNF levels of all our chronic pain patients were lower compared to the healthy control group.

With regard to the BDNF levels and immune cell numbers based on anxiety scores in the different chronic pain diseases, a significant difference was found exclusively in the level of free BDNF in OA patients. There does not seem to be a study investigating BDNF and anxiety scores in patients suffering with OA, but there are several studies on BDNF and mood disorders [62]. As mentioned above, anxiety is a common co-morbid of OA [63]. Furthermore, it is important to know that BDNF influences the human mood [64].

This study has some limitations. The ratio between females and males was not equal among the groups. Most of the patients were female, especially in the group of CWP patients. However, this reflects the prevalence of these groups of patients [65]. As mentioned above, this gender-related issue should be considered for future studies. Daily activity, including exercise, could also influence the level of BDNF [28,66]. Additionally, we did not record the full medications and nonpharmacological treatments in these patients. It has been reported that both pharmacological and nonpharmacological interventions can influence the level of BDNF [19,67,68]. Furthermore, nutrition and body composition parameters have been shown to influence the level of BDNF, as well [69,70]. Therefore, in future studies, it would be of importance to include these information in the analyses.

## 5. Conclusions

A general conformity between the pathomechanisms of different chronic pain diseases seems to exist, although there are unique findings only in specific chronic pain groups, as observed in BDNF and analyzed immune cells, particularly NK cells. Our results also highlight that the wider the region of the pain, the higher severity (pain, anxiety, and depression) the patients experience.

## Figures and Tables

**Table 1:** Age, sex, and body-related parameters in healthy subjects, osteoarthritis, chronic low back pain, and chronic widespread pain patients.

Parameter	HS	OA	cLBP	CWP	*p*-Value
	Mean ± SEM	Mean ± SEM	Mean ± SEM	Mean ± SEM	
Age (years)	49.97 ± 1.97	55.86 ± 1.91	52.24 ± 2.34	55.92 ± 1.28	0.076
Sex (N; male/female)	10/25	15/22	12/26	3/34	0.014 ^§^
Pain (VAS)	0.03 ± 0.03	5.43 ± 0.19	5.88 ± 0.20	6.39 ± 0.28	0.00
Height (cm)	169.33 ± 1.42	173.05 ± 1.57	170.39 ± 1.20	166.59 ± 1.09	0.008
Body weight (kg)	71.69 ± 2.96	88.06 ± 3.91	77.75 ± 2.81	77.40 ± 3.17	0.006
BMI (kg/cm^2^)	24.77 ± 0.76	29.14 ± 1.03	26.70 ± 0.86	27.68 ± 0.92	0.008
SMM (kg)	28.76 ± 1.20	32.37 ± 1.40	28.49 ± 0.92	26.90 ± 0.89	0.006
BFM (kg)	19.91 ± 1.71	29.97 ± 2.29	26.17 ± 2.10	28.54 ± 1.89	0.004
BWM (kg)	37.97 ± 1.45	42.65 ± 1.72	37.88 ± 1.12	35.87 ± 1.07	0.005
FFM (kg)	51.78 ± 1.97	58.09 ± 2.34	51.58 ± 1.52	48.60 ± 1.45	0.004
BFP (%)	27.10 ± 1.33	33.18 ± 1.40	32.21 ± 1.72	35.74 ± 1.06	0.000
WHR	0.92 ± 0.01	0.99 ± 0.01	0.97 ± 0.02	0.98 ± 0.01	0.001

Note: HS: Healthy subject; OA: Osteoarthritis; cLBP: chronic low back pain; CWP: Chronic widespread pain; VAS: Visual Analogue Scale; BMI: body mass index; SMM: skeletal muscle mass; BFM: body fat mass; BWM: body water mass; FFM: fat free mass; BFP: body fat percentage; WHR: waist–hip ratio. ANOVA test was performed for age and body composition-related parameters; ^§^ chi-square test.

**Table 2:** Immune cell profiles in HSs and OA, cLBP, and CWP patients.

	HS (*N* = 35)	OA (*N* = 37)	cLBP (*N* = 38)	CWP (*N* = 37)	*p*-Value
	Median (IQR)	Median (IQR)	Median (IQR)	Median (IQR)	
CD3^+^ (number of cells)	1390.00 (1023.00–1671.00)	1206.00 (931.00–1436.50)	1219.50 (1066.25–1716.75)	1305.00 (1112.50–1573.50)	N.S
CD3^+^ CD4^+^ (number of cells)	865.00 (675.00–1171.00)	787.00 (545.00–1043.00)	859.50 (703.50–1080.25)	889.00 (732.00–1064.00)	N.S
CD3^+^ CD8^+^ (number of cells)	452.00 (309.00–579.00)	386.00 (254.50–494.00)	385.50 (272.25–564.25)	426.00 (284.00–510.00)	N.S
CD8^+^ (number of cells)	628.00 (466.00–737.00)	507.00 (364.00–686.00)	557.50 (372.00–763.25)	527.00 (423.00–658.50)	N.S
CD4^−^ CD8^−^ T cells (number of cells)	32.00 (21.00–59.00)	29.00 (18.50–61.00)	37.00 (28.25–61.00)	33.00 (24.00–67.50)	N.S
CD4^+^ CD8^+^ T cells (number of cells)	22.00 (13.00–37.00)	16.00 (9.00–27.50)	15.00 (7.00–27.75)	14.00 (9.00–28.00)	N.S
CD3^+^ CD20^+^ (number of cells)	58.00 (37.00–94.00)	52.00 (32.00–79.50)	45.50 (28.00–91.00)	38.00 (25.50–66.50)	N.S
CD3^+^ CD56^+^ (number of cells)	97.00 (36.00–141.00)	63.00 (38.50–140.50)	78.00 (44.00–183.25)	112.00 (36.00–191.50)	N.S
CD3^−^ CD56_dim_ (number of cells)	262.00 (236.00–347.00)	226.00 (152.50–320.50)	241.50 (152.50–391.00)	215.00 (153.50–359.50)	N.S
CD3^−^ CD56_bright_ (number of cells)	16.00 (9.00–22.00)	11.00 (7.50–17.50)	13.00 (8.00–17.00)	13.00 (8.00-16.00)	0.03
CD3^−^ CD56^+^ CD8^+^ (number of cells)	287.00 (247.00–356.00)	288.00 (165.00–328.00)	256.00 (166.00–398.25)	224.00 (167.00–370.50)	N.S
CD20^+^ CD3^−^ (number of cells)	243.00 (180.00–302.00)	180.00 (121.00–228.00)	192.50 (122.75–281.50)	184.00 (140.00–260.00)	N.S

Note: OA: osteoarthritis; cLBP: chronic low back pain; CWP: chronic widespread pain; N.S: Not significant.

**Table 3:** Subgroup of patients based on HADS-A and HADS-D scores (% of patients).

	HADS-A Score		
	≤7	8–10	≥11
OA	81.1%	8.1%	10.8%
cLBP	71.1%	10.5%	18.4%
CWP	43.2%	21.7%	35.1%
	**HADS-D Score**		
	**≤** **7**	**8–10**	**≥** **11**
OA	94.6%	0.0%	5.4%
cLBP	71.1%	10.5%	18.4%
CWP	56.8%	16.2%	27.0%

Note: HADS-A/D: Hospital Anxiety and Depression Scale-Anxiety/Depression; OA: osteoarthritis; cLBP: chronic low back pain; CWP: chronic widespread pain.

**Table 4:** Correlation of clinical data and biological mediators in OA, cLBP, and CWP patients.

	Clinical Parameters	Clinical Biomediator Parameters	Correlation and *p*-Value	
OA	BW	BNDF Free	R: −0.357; *p =* 0.030	
	BW	BDNF Total	R: −0.479; *p =* 0.003	
	HADS-D		CD3^−^ CD56_bright_	R: −0.395; *p =* 0.015
cLBP	Pain (VAS)	NADS-A		R: 0.532; *p =* 0.001
		NADS-D	R: 0.595; *p =* 0.000	
CWP	Pain (VAS)	HADS-A		R: 0.328; *p =* 0.047
		HADS-D		R: 0.328; *p =* 0.047

Note: HADS-A/D: Hospital Anxiety and Depression Scale-Anxiety/Depression; OA: osteoarthritis; cLBP: chronic low back pain; CWP: chronic widespread pain; BW: body weight; VAS: Visual Analogue Scale.

## Data Availability

The datasets used and/or analyzed during the present study are available from the corresponding author on reasonable request.

## Supplementary Materials

The following are available online at https://www.mdpi.com/article/10.3390/medicina57040327/s1, Table S1: BDNF levels and immune cell numbers based on anxiety score in OA, cLBP, and CWP patients; Table S2: BDNF levels and immune cell numbers based on depression score in OA, cLBP, and CWP patients; Table S3: Level of BDNF and immune cells in all patient group based on different anxiety and depression scores.
