# Supplementary material for: Brain-Derived Neurotrophic Factor and Immune Cells in Osteoarthritis, Chronic Low Back Pain, and Chronic Widespread Pain Patients: Association with Anxiety and Depression
Source: Medicina (Kaunas). 2021 Apr 1;57(4):327. doi: 10.3390/medicina57040327 (PMC8065931; doi:10.3390/medicina57040327)
Supplement: Supplementary file 1 [file medicina-57-00327-s001.pdf]

Supplementary Material

Table 1. BDNF levels and immune cell numbers based on anxiety score in OA, cLBP, and CWP

		**BDNF Free** (ng/ml)		**BDNF Total** (ng/ml)		**CD3⁻CD56bright** (number of cells)		**CD20⁺CD3⁻** (number of cells)	
Group	HADS-A Score	Median (min-max)	p	Median (min-max)	p	Median (min-max)	p	Median (min-max)	p
OA	≤ 7 (N=30)	16.12 (2.66-30.79)	0.023	26.60 (1.11-51.30)	N.S	53.00 (0.00-197.00)	N.S	12.00 (0.00-45.00)	N.S
	8-10 (N=3)	21.47 (13.04–30.08)		30.13 (21.71-30.86)		77.00 (52.00-82.00)		10.00 (8.00-16.00)	
	≥ 11 (N=4)	25.80 (19.41-33.35)		41.48 (34.90-59.14)		39.50 (26.00-66.00)		8.00 (2.00-14.00)	
cLBP	≤ 7 (N=27)	19.63 (1.67-44.27)	N.S	31.74 (0.18-48.04)	N.S	53.00 (4.00-149.00)	N.S	13.00 (5.00-30.00)	N.S
	8-10 (N=4)	9.79 (2.46-22.56)		16.41 (3.35-29.45)		39.50 (28.00-97.00)		9.50 (6.00-19.00)	
	≥ 11 (N=7)	16.13 (7.82-49.89)		27.02 (9.47-48.31)		28.00 (9.00-188.00)		14.00 (7.00-17.00)	
CWP	≤ 7 (N=16)	19.57 (5.96-35.92)	N.S	34.46 (9.99-64.26)	N.S	36.50 (8.00-79.00)	N.S	13.00 (2.00-24.00)	N.S
	8-10 (N=8)	19.86 (8.25-45.23)		26.78 (0.49-54.78)		28.00 (6.00-89.00)		13.00 (8.00-27.00)	
	≥ 11 (N=13)	26.68 (4.14-37.84)		28.43 (3.66-59.09)		59.00 (8.00-203.00)		10.00 (5.00-33.00)	

HADS-A/D: Hospital Anxiety and Depression Scale-Anxiety/Depression; OA: Osteoarthritis; cLBP: Chronic low back pain; CWP: Chronic widespread pain; N.S: Not significant.

Table 2. BDNF levels and immune cell numbers based on depression score in OA, cLBP, and CWP

		**BDNF Free** (ng/ml)		**BDNF Total** (ng/ml)		**$CD3^-CD56_{bright}$** (number of cells)		**$CD20^+CD3^-$** (number of cells)	
Group	HADS-D Score	Median (Min-Max)	p	Median (Min-Max)	p	Median (Min-Max)	p	Median (Min-Max)	p
OA	≤ 7 (N=35)	16.66 (2.66-33.35)	N.S	30.13 (1.11-59.14)	N.S	51.00 (0.00-197.00)	N.S	12.00 (0.00-45.00)	N.S
	8-10 (N=0)	--		--		--		--	
	≥ 11 (N=2)	22.43 (19.41-25.44)		35.98 (34.90-37.06)		63.00 (6.00-66.00)		9.00 (7.00-11.00)	
cLBP	≤ 7 (N=27)	19.08 (1.67-44.27)	N.S	31.74 (0.18-48.04)	N.S	53.00 (4.00-176.00)	N.S	13.00 (5.00-30.00)	0.027
	8-10 (N=4	21.06 (16.13-29.42)		28.24 (22.16-42.54)		24.50 (9.00-28.00)		11.00 (6.00-29.00)	
	≥ 11 (N=7)	14.59 (5.06-49.89)		23.91 (6.02-48.31)		43.00 (16.00-188.00)		16.00 (7.00-23.00)	
CWP	≤ 7 (N=21)	18.70 (5.96-38.21)	N.S	25.40 (0.49-54.78)	N.S	35.00 (6.00-203.00)	N.S	12.00 (3.00-29.00)	N.S
	8-10 (N=6)	27.23 (4.14-37.84)		39.56 (7.04-64.26)		38.50 (23.00-79.00)		13.50 (8.00-24.00)	
	≥ 11 (N=10)	28.13 (7.98-45.23)		39.86 (3.66-59.09)		66.00 (10.00-91.00)		13.00 (2.00-33.00)	

HADS-A/D: Hospital Anxiety and Depression Scale-Anxiety/Depression; OA: Osteoarthritis; cLBP: Chronic low back pain; CWP: Chronic widespread pain; N.S: Not significant.

Table 3. Level of BDNF and immune cells in all patient groups based on different anxiety and depression scores

	**HADS-A Score**			**p**
	**≤ 7**	**8 – 10**	**≥ 11**	
	Median (Min-Max)			
BDNF Free (ng/ml)	18.57 (13.40 – 25.87)	18.91 (8.84 – 29.12)	19.48 (9.54 – 31.82)	0.210
BDNF Total (ng/ml)	30.91 (21.15 – 38.32)	26.8 (7.20 – 30.86)	33.22 (15.69 – 47.20)	0.099
$CD3^{-}$ $CD56_{bright}$ (number of cells)	13.00 (8.00 – 18.00)	12.00 (8.00 – 16.00)	10.00 (7.25 – 15.75)	0.959
$CD20^{+}CD3^{-}$ (number of cells)	168.00 (119.00 – 254.00)	224.00 (150.00 – 289.00)	198.00 (148.50 – 278.25)	0.184

	**HADS-D Score**			**p**
	**≤ 7**	**8 – 10**	**≥ 11**	
	Median (Min-Max)			
BDNF Free (ng/ml)	17.38 (11.63 – 25.61)	21.06 (17.63 – 35.90)	19.41 (14.59 – 32.80)	0.249
BDNF Total (ng/ml)	28.17 (19.26 – 37.70)	32.12 (25.81 – 49.74)	34.55 (20.59 – 47.35)	0.692
$CD3^{-}$ $CD56_{bright}$ (number of cells)	12.00 (8.00 – 17.00)	13.50 (8.00 – 17.25)	13.00 (7.00 – 17.00)	0.682
$CD20^{+}CD3^{-}$ (number of cells)	171.00 (122.00 – 244.00)	241.00 (187.75 – 282.25)	217.00 (139.00 – 297.00)	0.101

HADS-A/D: Hospital Anxiety and Depression Scale-Anxiety/Depression; OA: Osteoarthritis; cLBP: Chronic low back pain; CWP: Chronic widespread pain; N.S: Not significant.
